# The use of routinely collected electronic prescribing data to benchmark intravenous antibiotic use between two tertiary paediatric haematology-oncology inpatient units: a retrospective study

**DOI:** 10.1093/jacamr/dlad142

**Published:** 2023-12-28

**Authors:** Samuel Channon-Wells, Caroline Hérin, Ismail Elbeshlawi, Juliet Gray, Sanjay Patel, Stephane Paulus

**Affiliations:** Department of Paediatrics, Oxford University Hospitals NHS Foundation Trust, Oxford, UK; Department of Infectious Disease, Imperial College London, London, UK; School of Medicine, St Mary’s Hospital, Praed Street London W2 1NY, UK; Department of Paediatrics, Oxford University Hospitals NHS Foundation Trust, Oxford, UK; Department of Paediatrics, Oxford University Hospitals NHS Foundation Trust, Oxford, UK; Department of Paediatric Infectious Diseases and Immunology, University Hospital Southampton NHS Foundation Trust, Tremona Road, Southampton SO16 6YD, UK; Centre for Cancer Immunology, University of Southampton and Department of Paediatric Oncology, University Hospital Southampton NHS Foundation Trust, Southampton SO17 1BJ, UK; Department of Paediatric Infectious Diseases and Immunology, University Hospital Southampton NHS Foundation Trust, Tremona Road, Southampton SO16 6YD, UK; Department of Paediatrics, Oxford University Hospitals NHS Foundation Trust, Oxford, UK

## Abstract

**Background:**

High-quality systematic data on antimicrobial use in UK inpatient paediatric haematology-oncology services are lacking, despite this population being at high risk from antimicrobial exposure and resistance.

**Objectives:**

We conducted a retrospective study to demonstrate how routinely collected electronic prescribing data can address this issue.

**Patients and methods:**

This retrospective study describes and compares IV antibiotic consumption between two UK paediatric haematology-oncology inpatient units, between 2018 and 2022. Both sites provide similar services and receive proactive antimicrobial stewardship input. Data were extracted from each site’s antimicrobial surveillance system, which report monthly days of therapy (DOT) per 100 patient-days (PD). Consumption was reported for specific and total antibiotics. Trends were modelled using linear regression and autoregressive moving average models.

**Results:**

Total IV antibiotic consumption at each site was similar. Median monthly DOT per 100 PD were 25.9 (IQR: 22.1–34.0) and 29.4 (24.2–34.9). Total antibiotic use declined at both sites, with estimated annual yearly reductions of 3.52 DOT per 100 PD (95% CI: 0.46–6.59) and 2.57 (1.30–3.85). Absolute consumption was similar for carbapenems, piperacillin/tazobactam and aminoglycosides, whilst ceftriaxone and teicoplanin demonstrated approximately 3-fold relative differences in median monthly consumption. Meropenem, piperacillin/tazobactam, teicoplanin, vancomycin and gentamicin all demonstrated statistically significant reductions in use over time at either one or both sites, although this was most marked for piperacillin/tazobactam and vancomycin.

**Conclusions:**

Routinely collected electronic prescribing data can aid benchmarking of antibiotic use in paediatric haematology-oncology inpatients, highlighting areas to target stewardship strategies, and evaluating their impact. This approach should be rolled out nationally, and to other high-risk groups.

## Introduction

Antimicrobial consumption (AC) in children presenting or admitted to hospital is high, both in the UK and globally, and contributes to antimicrobial resistance (AMR).^1^ High AC and AMR are a particular concern in paediatric haematology and oncology specialties.^2^ AMR and AC vary by centre,^3^ highlighting the importance of accurate and systematic surveillance.

Unfortunately, major issues exist in the methods used for measuring and reporting AC in children. These include the standard use of the inappropriate DDD metric,^4,5^ estimations from dispensary data rather than actual patient-level administrations^6^ and lack of paediatric-specific reporting.^7,8^ This is a major barrier to benchmarking AC and sharing best antimicrobial stewardship (AMS) practices between centres and regions.

Point-prevalence surveys are often used to collect patient-level AC data in children.^3,8^ These manual surveys are valuable tools to assess appropriateness of antimicrobial prescriptions, but are labour intensive, and only provide a snapshot in time, resulting in poor estimates of trends over time.^5^ Continuous data from electronic prescribing systems have been shown to improve reliability of estimating AC over time, enhancing the ability to monitor trends and the impact of AMS programmes, but are currently underutilized.

The inevitable move towards electronic prescribing in the UK NHS and other high-income countries presents an opportunity to establish more systematic approaches to benchmarking AC in key paediatric populations. Electronic prescribing systems allow application of the days of therapy (DOT) metric, which is theoretically and practically more suitable for paediatric populations. To demonstrate this, we performed a retrospective study comparing AC between haematology-oncology units at two UK centres.

## Materials and methods

### Ethics

Permission to use anonymous aggregated data for benchmarking purposes was obtained through Oxford University Hospitals (OUH) and University Hospital Southampton (UHS) NHS Foundation Trusts as part of two ongoing quality improvement projects.

### Design and setting

This retrospective analysis compares data from the inpatient paediatric haematology and oncology units at two UK university hospitals. Each hospital provides tertiary-level haematology and oncology services to populations of approximately 3–4 million people and treats around 100–120 children with new oncological diagnoses yearly. Both deliver standard chemotherapy and autologous stem cell transplants in line with national/international protocols. Neither site undertakes allogeneic paediatric stem cell transplants. Specialist paediatric infectious diseases and AMS programmes are available at each centre, providing consult-based services and routine review of all children on antimicrobials through regular AMS rounds (2–3 times per week).

### Data collection

Both hospitals have paediatric antimicrobial surveillance systems (see Supplementary Methods, available as Supplementary data at  *JAC-AMR*  Online), reporting aggregated ward-level DOT for all antimicrobials, calculated directly from electronic health records, corrected for case load using the patient-days (PD) denominator: defined as the number of inpatients at midnight each day.

Between January 2018 and December 2022, monthly DOT per 100 PD for each unit were extracted directly from respective AMS systems for a subgroup of IV antibiotics, selected by clinical consensus based on their relevance to antibiotic prescribing in current haematology-oncology guidelines. Total antibiotic use was calculated as the sum of these antibiotics. Authors did not have direct access to the raw databases from which aggregated data were extracted.

### Statistical methods

Consumption was reported as median (IQR) monthly DOT per 100 PD. Median estimates were compared using the Wilcoxon rank-sum test. Time series were modelled using linear regression or autoregressive moving-average models, including seasonality terms, with final model choice based on auto correlation function (ACF) and partial-ACF plots, and optimal Akaike information criterion.^9^ Estimates of change in consumption over time were reported with 95% CI and *P* values using robust standard errors where appropriate,^10^ without multiple hypothesis correction. Statistical analysis was performed using R version 4.1.1.^11^

## Results

**Figure 1.**
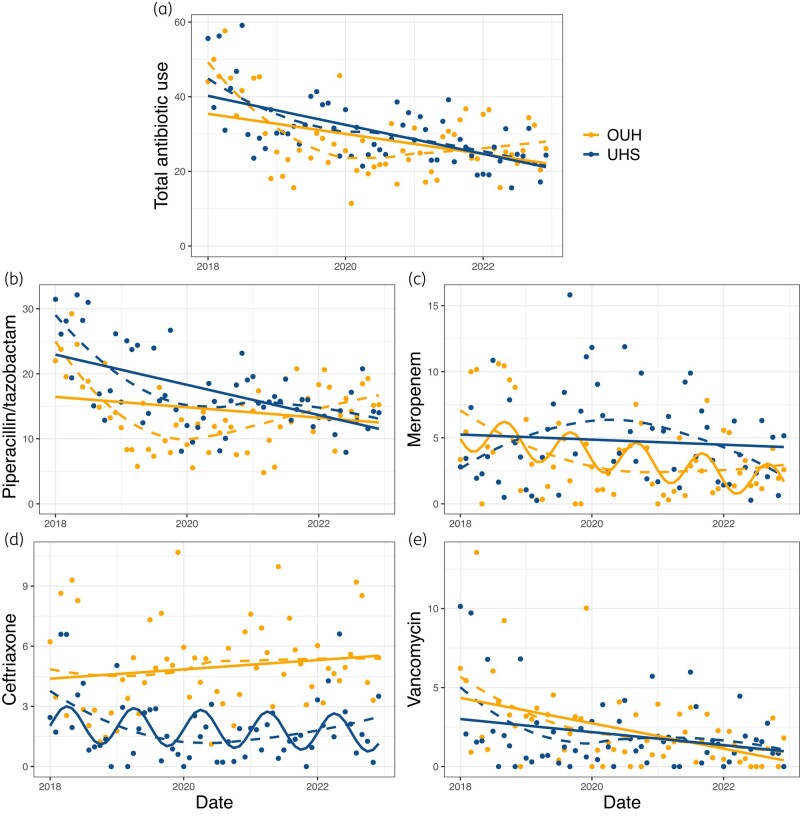
IV antibiotic use by site in DOT per 100 PD over the study period. (a) Total antibiotic use, (b) piperacillin/tazobactam, (c) meropenem, (d) ceftriaxone, (e) vancomycin. Dots represent raw monthly DOT per 100 PD. Solid lines represent fitted models for estimating trends over time, as described in the methods, dashed lines represent LOESS-fitted curves to demonstrate locally averaged changes. Apparent seasonal trends depicted by solid sinusoidal curves for meropenem (OUH) and ceftriaxone (UHS) are statistical artefacts resulting from applying different seasonality terms to model each dataset (see methods for details). OUH, Oxford University Hospitals; UHS, University Hospital Southampton.

Over the 5 year period total PD were 18 505 for OUH and 24 427 for UHS. Total IV antibiotic consumption was similar by site, with median monthly consumption of 25.9 (OUH) and 29.4 (UHS) DOT per 100 PD (*P* = 0.19, Table 1). We observed similar annual decreases in total consumption over time for both sites (Figure 1a, Table S1).

**Table 1.** IV consumption by antibiotic across the entire study period, displayed as median and IQR for monthly DOT per 100 PD. *P* values are calculated using the Wilcoxon rank-sum (WRS) test to compare median consumption over the entire study period

	OUH		UHS		WRS test *P* value
Antimicrobial	Median	IQR	Median	IQR	
All	25.87	22.09–33.94	29.37	24.20–34.91	0.19
Amikacin	0.00	0.00–0.00	0.00	0.00–0.00	0.33
Gentamicin	0.55	0.26–1.28	0.78	0.16–1.84	0.38
Ceftazidime	0.00	0.00–0.00	0.00	0.00–0.05	0.92
Ceftriaxone	4.90	3.27–5.97	1.54	0.79–2.67	<0.0001
Ciprofloxacin	0.00	0.00–0.46	0.00	0.00–1.39	0.13
Ertapenem	0.00	0.00–0.00	0.00	0.00–0.00	0.090
Flucloxacillin	0.00	0.00–0.45	0.00	0.00–0.62	0.54
Meropenem	2.82	1.13–4.93	3.65	2.03–6.77	0.027
Piperacillin/tazobactam	13.82	9.76–17.90	15.77	13.16–19.43	0.0057
Teicoplanin	0.53	0.00–2.44	1.69	0.48–2.66	0.0083
Vancomycin	1.65	0.63–3.28	1.49	0.59–2.28	0.38

IQR, Interquartile Range; OUH, Oxford University Hospitals; UHS, University Hospital Southampton.

### Usage by antibiotic

Median monthly DOT per 100 PD was significantly greater at UHS versus OUH for meropenem, piperacillin/tazobactam and teicoplanin (Table 1), although the relative differences between sites were small for meropenem and piperacillin/tazobactam. Ceftriaxone use was three times lower at UHS (Table 1)—a pattern seen across the entire study period (Figure 1d). Other antibiotics were used less frequently, with no significant difference between sites (Table 1, Figure S1). Monthly mean, standard deviation and percentage of total antibiotic use are presented in Table S2.

### Trends for specific antibiotics

For the UHS site, piperacillin/tazobactam consumption decreased annually by 2.3 DOT per 100 PD (95% CI: 1.3–3.3) (Figure 1b, Table S1). A decrease was also seen at OUH, although this was not statistically significant (annual decrease 0.8 DOT per 100 PD, 95% CI: −1.73 to 3.33). Locally estimated scatterplot smoothing (LOESS)-fitted curves show that consumption of piperacillin/tazobactam at OUH decreased in 2018, with steady usage for subsequent years. Conversely, meropenem consumption showed a significant decrease over time at the OUH site, and no change at UHS (Figure 1c, Table S1). Ceftriaxone consumption showed no significant change over time at either site (Figure 1d, Table S1).

A small but statistically significant decrease in vancomycin consumption was observed at both sites, with a greater decrease at OUH than UHS (annual decreases of 0.8 and 0.4 DOT per 100 PD respectively, Table S1, Figure 1e). Changes in teicoplanin use were small, and only statistically significant for OUH. Similarly, we observed a small decrease in gentamicin use at both sites, which was significant only for UHS.

## Discussion

This retrospective study sought to describe IV antibiotic use at two inpatient paediatric haematology-oncology units. Our findings demonstrate substantial similarity in overall use and trends between sites. Key strengths of this approach are the simplicity, use of longitudinal administration data rather than dispensary data or point-prevalence surveys, and paediatric-appropriate metrics. The observed decrease in overall antibiotic use at both sites is a welcome finding, given the worrying global pattern of increased AMR rates in children and adults.^12^ The very low rate of aminoglycoside and glycopeptide consumption is also reassuring, suggesting good adherence to national guidelines on neutropenic sepsis management.^13^

Although prescribing patterns at both sites were similar overall, significant differences were observed, demonstrating the power of this approach to produce vital benchmarking data. For example, the observed lower rate of ceftriaxone use at UHS identifies a possible target for AMS interventions. As very broad-spectrum antimicrobial use is high in this vulnerable patient group, especially piperacillin/tazobactam and meropenem, a switch to ceftriaxone when appropriate, such as in non-neutropenic febrile patients, is desirable, and also facilitates ambulatory care. Targeting paediatric haematology-oncology AMS interventions to specific antimicrobial groups has been shown to be feasible and safe whilst also reducing antimicrobial exposure, AMR rates and cost.^14,15^ Our use of continuous data also enables temporal intrasite benchmarking, and could be used to assess the impact of such targeted interventions, with greater precision than other methods.

Lack of similar data limits comparisons that can be made between our data and those of other centres. Where similar data are available, there are often multiple confounders limiting interpretability.^3,16^ This highlights the need for more systematic approaches to AC reporting in children. Numerous expert consensus reviews have highlighted the importance of systematic surveillance of both AC and AMR,^17,18^ as understanding the interplay between these factors is crucial to developing effective and safe AMS strategies.

Our study has important limitations. Including only two sites, neither of which offers allogenic BMT, limits generalizability. However, this is a highly specialized service, offered at a small number of centres. Our data will therefore be meaningful to many units in the UK and abroad.

In this analysis we were unable to link data to report additional measures of interest, including length of antibiotic course, dosing, indication, prophylaxis versus treatment, case mix, or clinical outcomes. These additions would certainly provide richer data, but are more complex to automate, so are currently not feasible for most paediatric AMS services in the UK, including our own, many of which have no dedicated funding.^19^ We also restricted this analysis to IV antibiotics to present a clear message. Future work will focus on additional antimicrobial groups and routes. We have assumed the case mix is comparable based on the scope and size of each service, and through anecdotal discussion with service providers. However, potential differences remain that could affect our results, but which we could not directly measure, such as the proportions of patients with haematological or oncological conditions treated outside these specialist wards.

Despite these limitations, we believe our study establishes a simple and reproducible methodology for intrasite and intersite benchmarking of AC. Our method uses routinely collected electronic prescribing data, and reports more accurate and reliable measures of AC than current reporting metrics in the UK. We show that this approach can highlight clinically impactful similarities and differences in practice. This provides a template for coordinating future large-scale paediatric AC monitoring in hospitals. Such programmes would fill the existing void in systematic data on paediatric AC, but would require a centrally coordinated approach to enable uniformity of data collection, analysis and reporting, to maximize comparability. This highlights the crucial role played by national bodies in recognizing the risk of AMR in children and funding paediatric AMS services appropriately.^19,20^

## Supplementary Material

dlad142_Supplementary_Data

## Supplementary data

Supplementary Methods, Figure S1 and Tables S1 and S2 are available as Supplementary data at *JAC-AMR* Online.

## References

[1] Hagedoorn NN, Borensztajn DM, Nijman R et al Variation in antibiotic prescription rates in febrile children presenting to emergency departments across Europe (MOFICHE): a multicentre observational study. PLoS Med 2020; 17: e1003208. 10.1371/journal.pmed.1003208 32813708 PMC7444592

[2] Wolf J, Sun Y, Tang L et al Antimicrobial stewardship barriers and goals in pediatric oncology and bone marrow transplantation: a survey of antimicrobial stewardship practitioners. Infect Control Hosp Epidemiol 2016; 37: 343–7. 10.1017/ice.2015.295 26639441

[3] Papan C, Reifenrath K, Last K et al Antimicrobial use in pediatric oncology and hematology in Germany and Austria, 2020/2021: a cross-sectional, multi-center point-prevalence study with a multi-step qualitative adjudication process. Lancet Reg Health Eur 2023; 28: e100599. 10.1016/j.lanepe.2023.100599 PMC10173264 37180743

[4] Moehring RW, Anderson DJ, Cochran RL et al Expert consensus on metrics to assess the impact of patient-level antimicrobial stewardship interventions in acute-care settings. Clin Infect Dis 2017; 64: 377–83. 10.1093/cid/ciw787 27927866 PMC5241782

[5] Channon-Wells S, Kwok M, Booth J et al The use of continuous electronic prescribing data to infer trends in antimicrobial consumption and estimate the impact of stewardship interventions in hospitalized children. J Antimicrob Chemother 2021; 76: 2464–71. 10.1093/jac/dkab187 34109397 PMC8361331

[6] Ockfen S, Egle L, Sauter K et al Meropenem use in pediatric oncology—audit on indication, appropriateness and consumption comparing patient derived and pharmacy dispensing data. Klin Padiatr 2021; 233: 278–85. 10.1055/a-1481-8905 34261135

[7] UK Health Security Agency (UKHSA) . English surveillance programme for antimicrobial utilisation and resistance (ESPAUR) report. https://www.gov.uk/government/publications/english-surveillance-programme-antimicrobial-utilisation-and-resistance-espaur-report.

[8] ECDC . Antimicrobial consumption in the EU/EEA (ESAC-Net)—Annual Epidemiological Report for 2021. 2022. https://www.ecdc.europa.eu/en/publications-data/surveillance-antimicrobial-consumption-europe-2021.

[9] Diggle PJ . Time Series: A Biostatistical Introduction. 1st edn. Clarendon Press, 1995.

[10] Diggle PJ, Heagerty P, Liang K-Y et al Analysis of Longitudinal Data. 2nd edn. Oxford University Press, 2013.

[11] R Core Team . R: A Language and Environment for Statistical Computing. 2022. https://cran.r-project.org/.

[12] Antimicrobial Resistance Collaborators . Global burden of bacterial antimicrobial resistance in 2019: a systematic analysis. Lancet 2022; 399: 629–55. 10.1016/S0140-6736(21)02724-0 35065702 PMC8841637

[13] NICE . Neutropenic sepsis: prevention and management in people with cancer. Clinical guideline [CG151]. 2012, updated January 2020. https://www.nice.org.uk/guidance/cg151. 39960996

[14] Karandikar MV, Milliren CE, Zaboulian R et al Limiting vancomycin exposure in pediatric oncology patients with febrile neutropenia may be associated with decreased vancomycin-resistant *Enterococcus* incidence. J Pediatric Infect Dis Soc 2020; 9: 428–36. 10.1093/jpids/piz064 31603472 PMC7495906

[15] Horikoshi Y, Kaneko T, Morikawa Y et al The north wind and the sun: pediatric antimicrobial stewardship program combining restrictive and persuasive approaches in hematology-oncology ward and hematopoietic stem cell transplant unit. Pediatr Infect Dis J 2018; 37: 164–8. 10.1097/INF.0000000000001746 28827495

[16] Mantadakis E, Kopsidas I, Coffin S et al A national study of antibiotic use in Greek pediatric hematology oncology and bone marrow transplant units. Antimicrob Steward Healthc Epidemiol 2022; 2: e71. 10.1017/ash.2022.43 36483391 PMC9726537

[17] Pezzani MD, Carrara E, Sibani M et al White paper: bridging the gap between human and animal surveillance data, antibiotic policy and stewardship in the hospital sector-practical guidance from the JPIAMR ARCH and COMBACTE-MAGNET EPI-net networks. J Antimicrob Chemother 2020; 75 Suppl 2: ii20–32. 10.1093/jac/dkaa426 33280046 PMC7719407

[18] Cercenado E, Rodriguez-Bano J, Alfonso JL et al Antimicrobial stewardship in hospitals: expert recommendation guidance document for activities in specific populations, syndromes and other aspects (PROA-2) from SEIMC, SEFH, SEMPSPGS, SEMICYUC and SEIP. Enferm Infecc Microbiol Clin (Engl Ed) 2023; 41: 238–42. 10.1016/j.eimc.2022.05.005 36610836

[19] Kopsidas I, Vergnano S, Spyridis N et al A survey on national pediatric antibiotic stewardship programs, networks and guidelines in 23 European countries. Pediatr Infect Dis J 2020; 39: e359–e62. 10.1097/INF.0000000000002835 32773659

[20] Vergnano S, Bamford A, Bandi S et al Paediatric antimicrobial stewardship programmes in the UK’s regional children’s hospitals. J Hosp Infect 2020; 105: 736–40. 10.1016/j.jhin.2020.05.030 32454075

