# Supplementary material for: The use of routinely collected electronic prescribing data to benchmark intravenous antibiotic use between two tertiary paediatric haematology-oncology inpatient units: a retrospective study
Source: JAC Antimicrob Resist. 2023 Dec 28;6(1):dlad142. doi: 10.1093/jacamr/dlad142 (PMC10753649; doi:10.1093/jacamr/dlad142)
Supplement: dlad142_Supplementary_Data [file dlad142_supplementary_data.docx]

Supplementary Materials

Table of Contents

[Supplementary methods 2](#_Toc151411289)

[Reporting standards: 2](#_Toc151411290)

[Antimicrobial surveillance systems: 2](#_Toc151411291)

[Antibiotic selection: 2](#_Toc151411292)

[Additional modelling details: 2](#_Toc151411293)

[Supplementary Tables: 3](#_Toc151411294)

[Table S1 3](#_Toc151411295)

[Table S2 4](#_Toc151411296)

[Supplementary Figures: 5](#_Toc151411297)

[Figure S1 5](#_Toc151411298)

[References 6](#_Toc151411299)

[Appendix 1 – The RECORD statement 7](#_Toc151411300)

# Supplementary methods

## Reporting standards:

This report has been compiled based on the RECORD guidelines for reporting studies using routinely-collected health data ([Appendix 1](#_Appendix_1_–)).^1^

## Antimicrobial surveillance systems:

The UHS antimicrobial surveillance system uses a commercially available product, Triscribe Ltd©.^2^ The OUH system uses a customised interactive application built using R,^3^ version 4.1.1, and the R-shiny package.^4^ Days of Therapy (DOT) per-100 patient days were extracted from the respective antimicrobial surveillance system for each site.

## Antibiotic selection:

Antibiotics analysed were restricted to the following list, and to intravenous routes only, as enteral administrations accounted for a small proportion of overall antimicrobial use on these wards: Amikacin, Ceftazidime, Ceftriaxone, Ciprofloxacin, Ertapenem, Flucloxacillin, Gentamicin, Meropenem, Piperacillin/tazobactam, Teicoplanin, and Vancomycin.

## Additional modelling details:

DOT per-100-PD over time was modelled fitting either linear regression or autoregressive moving-average (ARMA) models, with choice depending on Auto Correlation Function (ACF) and partial-ACF plots.^5^ Generalized additive model plots of DOT per-100-PD over time were inspected to look for evidence of non-linearity on the log-odds scale.^6^ Seasonality was modelled using yearly and 6-monthly sine/cosine variables, with Akaike information criterion used to compare model fit and select final models. Time series of residuals were examined for evidence of autocorrelation, along with additional diagnostic plots depending on final selected model. These models were used for curve fitting and estimating change in consumption over time for antibiotics with non-zero median consumption. Estimates were reported with 95% confidence intervals (CI) and p-values estimated using robust standard errors where appropriate,^7^ without correction for multiple hypothesis testing. LOESS curves were fit to timeseries plots to aid visualisation only.

# Supplementary Tables:

## Table S1

Estimates of yearly change in consumption for Total and specific antibiotic use, in DOT per-100-PD, presented with 95% confidence intervals and p-values. Models used were either linear regression (lm) or autoregressive moving-average models (ARMA-X-Y: X indicated autoregressive order; Y indicates moving average order). Model selection procedures are described in methods. CI: Confidence interval; OUH: Oxford University Hospitals; UHS: University Hospital Southampton.

|  |  |  | **Seasonality term** | **Yearly change in consumption** | | |
| --- | --- | --- | --- | --- | --- | --- |
| **Antibiotic** | **Site** | **Model** |  | **Estimate** | **95% CI** | **p-value** |
| All | OUH | ARMA-0-1 | None | -2.70 | (-4.68, -0.71) | 0.0077 |
|  | UHS | ARMA-0-4 | None | -3.88 | (-5.60, -2.16) | <0.0001 |
| Ceftriaxone | OUH | LM | None | 0.23 | (-0.16, 0.62) | 0.25 |
|  | UHS | LM | Yearly | -0.09 | (-0.35, 0.17) | 0.51 |
| Gentamicin | OUH | LM | None | -0.19 | (-0.41, 0.03) | 0.091 |
|  | UHS | ARMA-0-5 | None | -0.14 | (-0.25, -0.03) | 0.014 |
| Meropenem | OUH | ARMA-0-1 | Yearly | -0.79 | (-1.35, -0.24) | 0.0051 |
|  | UHS | LM | None | -0.19 | (-0.81, 0.42) | 0.54 |
| Piperacillin/ tazobactam | OUH | ARMA-1-1 | None | -0.80 | (-3.33, 1.73) | 0.53 |
|  | UHS | ARMA-0-1 | None | -2.32 | (-3.32, -1.32) | <0.0001 |
| Teicoplanin | OUH | LM | None | -0.35 | (-0.65, -0.05) | 0.025 |
|  | UHS | ARMA-1-1 | None | -0.23 | (-0.50, 0.03) | 0.084 |
| Vancomycin | OUH | LM | None | -0.80 | (-1.21, -0.40) | 0.0003 |
|  | UHS | LM | None | -0.41 | (-0.79, -0.04) | 0.036 |

## Table S2

Intravenous consumption by antibiotic across the entire study period, displayed as mean and standard deviation (SD) for monthly DOT-per-100-PD, and percentage of total use by site. OUH: Oxford University Hospitals; UHS: University Hospital Southampton.

|  | **OUH** | | **UHS** | |
| --- | --- | --- | --- | --- |
| **Antibiotic** | **Mean (SD) DOT/100-PD** | **Percentage of total mean use** | **Mean (SD) DOT/100-PD** | **Percentage of total mean use** |
| Amikacin | 0.1 (0.3) | 0.3 | 0.0 (0.2) | 0.1 |
| Gentamicin | 1.0 (1.3) | 3.4 | 1.1 (1.1) | 3.5 |
| Ceftazidime | 0.4 (1.1) | 1.5 | 0.3 (0.7) | 1.0 |
| Ceftriaxone | 5.0 (2.2) | 17.2 | 1.9 (1.6) | 6.2 |
| Ciprofloxacin | 0.5 (1.2) | 1.8 | 1.0 (1.6) | 3.1 |
| Ertapenem | 0.1 (0.4) | 0.3 | 0.0 (0.0) | 0.0 |
| Meropenem | 3.5 (2.9) | 12.1 | 4.8 (3.5) | 15.6 |
| Piperacillin/ tazobactam | 14.0 (5.2) | 48.6 | 17.2 (6.0) | 56.4 |
| Teicoplanin | 1.4 (1.8) | 4.8 | 1.8 (1.3) | 5.9 |
| Vancomycin | 2.4 (2.6) | 8.3 | 2.0 (2.2) | 6.5 |
| Flucloxacillin | 0.5 (0.8) | 1.7 | 0.5 (0.9) | 1.6 |

# Supplementary Figures:

## Figure S1

Proportion of total antibiotic use for all included antimicrobials over time, separated by site. Specific antibiotics are grouped by class to aid visualisation. OUH: Oxford University Hospitals; UHS: University Hospital Southampton.


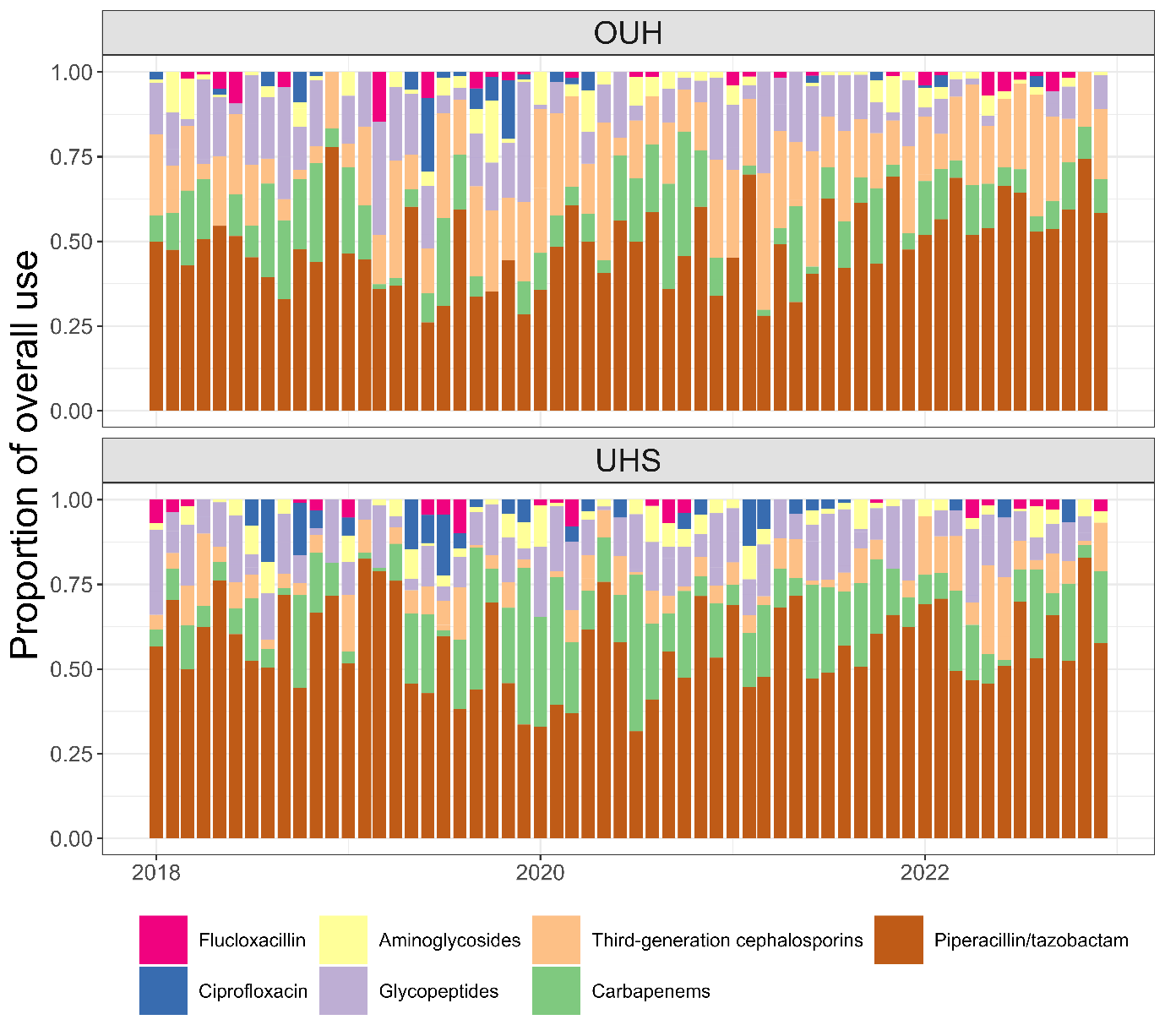


# References

1. Nicholls SG, Quach P, von Elm E, et al. The REporting of Studies Conducted Using Observational Routinely-Collected Health Data (RECORD) Statement: Methods for Arriving at Consensus and Developing Reporting Guidelines. *PLoS One* 2015; **10**(5): e0125620.

2. Triscribe©. 2023. https://www.triscribe.net/.

3. R Core Team (2022). R: A language and environment for statistical computing. R Foundation for Statistical Computing, Vienna, Austria.

4. Chang W, Cheng J, Allaire J, et al. shiny: Web Application Framework for R. R package version 1.7.4. 2022.

5. Diggle PJ. Time Series: A Biostatistical Introduction. 1st Edition ed. Oxford, UK: Oxford University Press; 1995.

6. Wood S. Generalized Additive Models: An Introduction with R.: Chapman & Hall; 2006.

7. Diggle PJ, Heagerty P, Liang K-Y, Zeger SL. Analysis of Longitudinal Data. Second Edition ed. Oxford, UK: Oxford University Press; 2013.

# Appendix 1 – The RECORD statement

**Checklist of items, extended from the STROBE statement, that should be reported in observational studies using routinely collected health data.**

|  | **Item No.** | **STROBE items** | **Location in manuscript where items are reported** | **RECORD items** | **Location in manuscript where items are reported** |
| --- | --- | --- | --- | --- | --- |
| **Title and abstract** | | | | | |
|  | 1 | (a) Indicate the study’s design with a commonly used term in the title or the abstract (b) Provide in the abstract an informative and balanced summary of what was done and what was found |  | RECORD 1.1: The type of data used should be specified in the title or abstract. When possible, the name of the databases used should be included.  RECORD 1.2: If applicable, the geographic region and timeframe within which the study took place should be reported in the title or abstract.  RECORD 1.3: If linkage between databases was conducted for the study, this should be clearly stated in the title or abstract. | PAGES 1,2  PAGE 2  NOT APPLICABLE |
| **Introduction** | | | | | |
| Background rationale | 2 | Explain the scientific background and rationale for the investigation being reported |  |  | PAGES 4-5 |
| Objectives | 3 | State specific objectives, including any prespecified hypotheses |  |  | PAGES 4-5 |
| **Methods** | | | | | |
| Study Design | 4 | Present key elements of study design early in the paper |  |  | PAGE 6 |
| Setting | 5 | Describe the setting, locations, and relevant dates, including periods of recruitment, exposure, follow-up, and data collection |  |  | PAGES 6-7 |
| Participants | 6 | *(a) Cohort study* - Give the eligibility criteria, and the sources and methods of selection of participants. Describe methods of follow-up  *Case-control study* - Give the eligibility criteria, and the sources and methods of case ascertainment and control selection. Give the rationale for the choice of cases and controls  *Cross-sectional study* - Give the eligibility criteria, and the sources and methods of selection of participants  *(b) Cohort study* - For matched studies, give matching criteria and number of exposed and unexposed  *Case-control study* - For matched studies, give matching criteria and the number of controls per case |  | RECORD 6.1: The methods of study population selection (such as codes or algorithms used to identify subjects) should be listed in detail. If this is not possible, an explanation should be provided.  RECORD 6.2: Any validation studies of the codes or algorithms used to select the population should be referenced. If validation was conducted for this study and not published elsewhere, detailed methods and results should be provided.  RECORD 6.3: If the study involved linkage of databases, consider use of a flow diagram or other graphical display to demonstrate the data linkage process, including the number of individuals with linked data at each stage. | PAGES 6-7  NOT APPLICABLE  NOT APPLICABLE |
| Variables | 7 | Clearly define all outcomes, exposures, predictors, potential confounders, and effect modifiers. Give diagnostic criteria, if applicable. |  | RECORD 7.1: A complete list of codes and algorithms used to classify exposures, outcomes, confounders, and effect modifiers should be provided. If these cannot be reported, an explanation should be provided. | PAGES 6-7,  SUPPLEMENTARY MATERIAL PAGE 2 |
| Data sources/ measurement | 8 | For each variable of interest, give sources of data and details of methods of assessment (measurement).  Describe comparability of assessment methods if there is more than one group |  |  | PAGE 6, SUPPLEMENTARY MATERIAL PAGE 2 |
| Bias | 9 | Describe any efforts to address potential sources of bias |  |  | PAGE 7 |
| Study size | 10 | Explain how the study size was arrived at |  |  | NOT APPLICABLE |
| Quantitative variables | 11 | Explain how quantitative variables were handled in the analyses. If applicable, describe which groupings were chosen, and why |  |  | PAGES 6-7 |
| Statistical methods | 12 | (a) Describe all statistical methods, including those used to control for confounding  (b) Describe any methods used to examine subgroups and interactions  (c) Explain how missing data were addressed  (d) *Cohort study* - If applicable, explain how loss to follow-up was addressed  *Case-control study* - If applicable, explain how matching of cases and controls was addressed  *Cross-sectional study* - If applicable, describe analytical methods taking account of sampling strategy  (e) Describe any sensitivity analyses |  |  | PAGE 7, SUPPLEMENTARY MATERIAL PAGE 2  NOT APPLICABLE  NOT APPLICABLE  NOT APPLICABLE  NOT APPLICABLE  NOT APPLICABLE  NOT APPLICABLE |
| Data access and cleaning methods |  | .. |  | RECORD 12.1: Authors should describe the extent to which the investigators had access to the database population used to create the study population.  RECORD 12.2: Authors should provide information on the data cleaning methods used in the study. | PAGE 7  NOT APPLICABLE |
| Linkage |  | .. |  | RECORD 12.3: State whether the study included person-level, institutional-level, or other data linkage across two or more databases. The methods of linkage and methods of linkage quality evaluation should be provided. | NOT APPLICABLE |
| **Results** | | | | | |
| Participants | 13 | (a) Report the numbers of individuals at each stage of the study (*e.g.*, numbers potentially eligible, examined for eligibility, confirmed eligible, included in the study, completing follow-up, and analysed)  (b) Give reasons for non-participation at each stage.  (c) Consider use of a flow diagram |  | RECORD 13.1: Describe in detail the selection of the persons included in the study (*i.e.,* study population selection) including filtering based on data quality, data availability and linkage. The selection of included persons can be described in the text and/or by means of the study flow diagram. | PAGE 8  NOT APPLICABLE  NOT APPLICABLE |
| Descriptive data | 14 | (a) Give characteristics of study participants (*e.g.*, demographic, clinical, social) and information on exposures and potential confounders  (b) Indicate the number of participants with missing data for each variable of interest  (c) *Cohort study* - summarise follow-up time (*e.g.*, average and total amount) |  |  | NOT APPLICABLE  NOT APPLICABLE  NOT APPLICABLE |
| Outcome data | 15 | *Cohort study* - Report numbers of outcome events or summary measures over time  *Case-control study* - Report numbers in each exposure category, or summary measures of exposure  *Cross-sectional study* - Report numbers of outcome events or summary measures |  |  | NOT APPLICABLE  NOT APPLICABLE  PAGES 8-9, AND TABLE 1, SUPPLEMENTARY TABLES 1-2 |
| Main results | 16 | (a) Give unadjusted estimates and, if applicable, confounder-adjusted estimates and their precision (e.g., 95% confidence interval). Make clear which confounders were adjusted for and why they were included  (b) Report category boundaries when continuous variables were categorized  (c) If relevant, consider translating estimates of relative risk into absolute risk for a meaningful time period |  |  | PAGES 8-9, AND TABLE 1, SUPPLEMENTARY TABLES 1-2  NOT APPLICABLE  NOT APPLICABLE |
| Other analyses | 17 | Report other analyses done—e.g., analyses of subgroups and interactions, and sensitivity analyses |  |  | NOT APPLICABLE |
| **Discussion** | | | | | |
| Key results | 18 | Summarise key results with reference to study objectives |  |  | PAGE 10,12 |
| Limitations | 19 | Discuss limitations of the study, taking into account sources of potential bias or imprecision. Discuss both direction and magnitude of any potential bias |  | RECORD 19.1: Discuss the implications of using data that were not created or collected to answer the specific research question(s). Include discussion of misclassification bias, unmeasured confounding, missing data, and changing eligibility over time, as they pertain to the study being reported. | PAGES 11 |
| Interpretation | 20 | Give a cautious overall interpretation of results considering objectives, limitations, multiplicity of analyses, results from similar studies, and other relevant evidence |  |  | PAGES 10,12 |
| Generalisability | 21 | Discuss the generalisability (external validity) of the study results |  |  | PAGES 11-12 |
| **Other Information** | | | | | |
| Funding | 22 | Give the source of funding and the role of the funders for the present study and, if applicable, for the original study on which the present article is based |  |  | PAGE 13 |
| Accessibility of protocol, raw data, and programming code |  | .. |  | RECORD 22.1: Authors should provide information on how to access any supplemental information such as the study protocol, raw data, or programming code. | NOT APPLICABLE |

*Reference: Benchimol EI, Smeeth L, Guttmann A, Harron K, Moher D, Petersen I, Sørensen HT, von Elm E, Langan SM, the RECORD Working Committee. The REporting of studies Conducted using Observational Routinely-collected health Data (RECORD) Statement. *PLoS Medicine* 2015; in press.

*Checklist is protected under Creative Commons Attribution ([CC BY](http://creativecommons.org/licenses/by/4.0/)) license.
